# New Appliances & Things Medical

**Published:** 1909-07-17

**Authors:** 


### NEW APPLIANCES & THINGS MEDICAL.

[We shall be glad to receive at our Office, 28 & 29 Southampton Street, Strand, London, W.C., from the manufacturers, specimens of all new preparations and appliances.]

### MILK STOUT.

(Mackeson and Co., Ltd., Ashford, Dover and Folkestone.)

We have received a sample of the above preparation which consists practically of good stout, containing, in addition to its ordinary carbo-hydrate content, 2.5 per cent. of milk sugar or lactose. We have so recently in the Special Hospital Commission on Beer and Stout drawn attention to the method of production, chemical and physiological properties of stout that we need only repeat here that it is a beverage of high nutritive value, important therapeutic characteristics, and good thirst-quenching properties. The above stout has had its nutritive value materially increased by the addition of 2½ per cent. of milk sugar. Lactose or milk sugar is the sugar occurring naturally in milk and possesses certain advantages of other sugars, one of which at any rate is that as such it is not fermentable by the ordinary fermentative agents. The addition of the lactose has not in any way altered the taste or palatability of the stout. This stout is produced by a patent process due to the ingenuity of Mr. Melhush. Lactose has unquestionably a diuretic action, and its presence in this quantity in stout imparts to the beverage a stimulating action upon the kidneys. Our investigations have led us to the conclusion that the stout in question actually is what it is represented to be, and in our opinion it will form a useful addition to the pleasant and nutritive beverages worthy of being recommended by medical men to their patients.

### ROYAL COLLEGE OF SURGEONS.

At the last quarterly meeting of the Council of the Royal College of Surgeons of England on July 8, under the chairmanship of Sir Henry Morris, president, the successful candidates at the recent election—Sir Watson Cheyne, Mr. R. Clement Lucas, and Mr. W. Harrison Cripps—took their seats as members of the Council. The President was congratulated by his colleagues on having received from his Majesty the honour of a Baronetcy. Mr. Henry T. Butlin, D.C.L., consulting surgeon to St. Bartholomew's Hospital, was elected President of the college, and Mr. A. Pearce Gould and Mr. R. Clement Lucas were elected Vice-Presidents. The following professors and lecturers were appointed for the ensuing year:—*Hunterian Professors.*—Messrs. Arthur Keith, W. Sampson Handley, George Coats, R. H. Paramore, and Charles Bolton. *Arris and Gale Lecturers.* Messrs. Sydney R. Scott and Peter Thompson. *Erasmus Wilson Lecturer.*—Mr. S. G. Shattock. *Arnott Demonstrator.*—Mr. Arthur Keith. An additional report from the Museum Committee regarding the revision of the Pathological Catalogue recommended the appointment, in addition to the present staff, of a pathological assistant and an expert to advise regarding the gynæcological specimens. It was agreed to carry out this proposal. The thanks of the council were accorded to the authorities of the Egypt Exploration Fund for a valuable gift of 83 VIth Dynasty skulls recently discovered at Abydos, in Egypt.

